# Evaluating the Impact of Casino-Shifts on Patient Flow in an Emergency Department: A Pilot Study

**DOI:** 10.7759/cureus.69713

**Published:** 2024-09-19

**Authors:** Melanie A Johnston, Rachel Goss, Kavish Chandra, Robert Goss, Paul Atkinson

**Affiliations:** 1 Emergency Medicine, Queen Elizabeth Hospital, Charlottetown, CAN; 2 Emergency Medicine, Saint John Regional Hospital, Dalhousie University, Saint John, CAN; 3 Anesthesiology, Saint John Regional Hospital, Dalhousie University, Saint John, CAN

**Keywords:** burnout, casino, emergency department, flow, patient outcomes, wellness

## Abstract

Objectives: Demand for emergency services has resulted in an increased number of physicians experiencing burnout. Burnout rates amongst emergency medicine physicians consistently exceed that of other specialties, with shift work being a large contributor to the phenomenon. Casino-shift scheduling has addressed this issue in several emergency departments (EDs). Casino-shift scheduling is modeled around circadian rhythm theory which shows that anchor sleep helps to preserve circadian rhythm and normalize sleep patterns. To account for this, casino-shift models schedule overnight coverage with two six-hour shifts, to allow both physicians to obtain some sleep within the 01:00 to 06:00 am anchor period. While the benefits of the model have been demonstrated concerning physician well-being, there is a paucity of evidence assessing if this model has any effect on quality measures such as patient flow. Given the importance of patient flows to ED functioning, the objective of our study was to determine the effect of a casino-shift trial on overnight patient flow variables in a tertiary ED.

Methods: We performed a retrospective analysis of administrative data for overnight (10:00 pm to 10:00 am) patient flow variables during a two-month casino-shift intervention period (September 9, 2019, to November 4, 2019) compared with a control period at an ED in Eastern Canada. We analyzed various measures of patient flow for patients presenting overnight between 10:00 pm to 10:00 am during the study period. Primary outcome measures were wait time (WT), length of stay (LOS), and admission rates.

Results: Of the 19170 patient visits, 16787 met inclusion criteria. The median overnight WTs for the casino-shift intervention period were longer at 68 minutes (interquartile range (IQR) 31-154 minutes), compared with 51 minutes (IQR 21-104 minutes) in the control group. The LOS for the intervention was 217 minutes (IQR 132-358 minutes) compared with 195 minutes (IQR 112-336 minutes) for the control. There were 166 admissions/month during the trial, and 193 admissions/month during the 10-month control period.

Conclusions: In our study, our patient flow indicators of median WT and LOS were longer in the casino-shift intervention period by an absolute difference of 17 minutes and 22 minutes respectively. As a relatively new concept in emergency medicine scheduling, the impact of the casino-shift model on both well-being and ED efficiency warrants further investigation given the potential benefits to an occupation prone to burnout.

## Introduction

Emergency physicians strive to provide high-quality, safe, and timely care for their patients. This has become increasingly difficult as our system faces a mismatch between patient demand and the emergency department's (ED's) ability to deliver care due to poor patient flow and departmental crowding [1]. Crowding has been correlated with a reduction in the quality of patient care, delays in assessment and treatment, and increased mortality [2].

The current demand for emergency care results in increased numbers of emergency physicians experiencing burnout and ultimately leaving the profession [3]. The high-stress environment, shift work, and long hours all contribute as stressors in the lives of emergency physicians [4,5]. The effects over time can be detrimental to health and those who are subject to symptoms of burnout are more likely to leave practice, retire early, or reduce their clinical hours [5-8]. A significant contributor to emergency physician burnout is sleep deprivation from the shift work model of scheduling.

To remedy the negative effects of shift work, many EDs have used the casino-shift framework. This scheduling strategy is modeled around circadian rhythm theory [6]. This theory shows that the drive to sleep is strongest between 0300 and 0500 and that any sleep obtained between 0100 and 0600, described as the anchor period, helps to preserve circadian rhythm, mimicking a more normal sleep pattern which helps to improve recovery time [5,6,9].

While the benefits of the casino-shift model have been demonstrated with regard to physician health and well-being, there is a notable gap in the literature assessing if this model has any effect on patient flow. Flow is defined by the Royal College of Emergency Medicine as "the passage of patients through the care pathway. It should be considered as a ‘home to home’ process, initiated when patients call for help from the community or present to a health care provider. It finishes when their episode of care is completed and they return home or to an appropriate care setting" [10].

Strategies studied to address the issue of crowding and flow in the ED have included doctor triage, rapid assessment areas, and tailoring physician schedules. Given the importance of patient flow to ED functioning, our goal was to evaluate the impact that a casino-shift trial had on patient flow by retrospectively analyzing administrative data during a two-month casino-shift trial.

## Materials and methods

Study design/setting

The Saint John Regional Emergency Department (SJRH ED) is a tertiary-level ED with an annual ED census of around 60,000 patients. The SJRH ED undertook a two-month “casino-shift” trial from September 9, 2019 to November 4, 2019. Under this model, the traditional eight-hour staggered overnight coverage shifts beginning at 3:00 pm, 4:00 pm, 6:00 pm, and 12:00 am were adjusted to include the casino-shift model of two six-hour split shifts for the 10:00 pm-10:00 am overnight coverage beginning at 10:00 pm and 4:00 am in addition to modified day and evening shifts (see Table 1).

**Table 1:** Shift times

Normal schedule	Casino-shift schedule
07:00 am-15:00 pm	Casino 2 04:00 am-10:00 am
08:00 am-16:00 pm	08:00 am-16:00 pm
08:30 am-16:00 pm	08:30 am-16:00 pm
12:00 pm-18:00 pm	10:00 am-18:00 pm
15:00 pm-23:00 pm	14:00 pm-22:00 pm
16:00 pm-24:00 pm	16:00 pm-24:00 pm
16:00 pm-14:00 pm	16:00 pm-24:00 pm
18:00 pm-02:00 am	18:00 pm-02:00 am
24:00 pm-08:00 am	Casino 1 22:00 pm-04:00 am

We conducted a retrospective analysis of patient flow indicators using administrative data for patients presenting to the ED overnight from 10:00 pm to 10:00 am. The study protocol was approved by the Horizon Health Network Research Ethics Board (approval 100909). We included a control group from the two-month periods before and after the trial (July 2019-December 2019), and the same six-month period in 2018 (July 2018-December 2018) in our data analysis.

Patient population

We included data on patients presenting overnight to the SJRH ED between 10:00 pm and 10:00 am during the study period. We excluded any visits that had incomplete data or missing information pertinent to the variables of interest.

Intervention

Using our electronic health record, we were able to reach and export data for patient attendance during the study window. This record enabled us to pull the patients' arrival time, time of physician assessment, departure time, and the number of admissions. A retrospective analysis of patient flow indicators using administrative data for patients who presented to the SJRH ED overnight during the study period, compared to a control period.

Outcome measures

Our primary patient flow indicators were wait time (WT), length of stay (LOS), and admission rates. We defined WT as the time from registration until the time a physician was assigned to the patient (door-to-physician time). LOS was defined as the time from registration until the time of discharge or admission to the hospital. Lastly, the number of patients admitted to the hospital included any patients who registered between 10:00 pm and 10:00 am and were admitted to the hospital during their visit to the ED.

Statistical analysis

The dataset was analyzed using Microsoft Excel (Microsoft® Corp., Redmond, WA, USA) to compile descriptive statistics of median and interquartile ranges. The data was analyzed as two subsets: overnight patient visits during the casino-shift intervention period (September 9 to November 4, 2019) and the control period of traditional scheduling (July 1 to December 29, 2018, and July 14 to September 9, November 4 to December 29, 2019). The median WT and LOS were calculated for each of these periods. Each of these groups was then further sub-analyzed to correlate with the casino-shift scheduling of 10:00 pm to 4:00 am, and 4:00 am to 10:00 am. The number of patient admissions in each broad grouping of casino-shift versus traditional scheduling periods was calculated.

## Results

A total of 19170 patient visits during the study period were reviewed. We excluded all visits with incomplete data and those felt to contain administrative recording errors (e.g. WT recorded > LOS); this resulted in the exclusion of 2383 patient visits. The dataset was analyzed as two subsets: overnight patient visits during the casino-shift intervention period (2495 patient visits), and the comparison period of overnight patient visits during traditional scheduling 14292 (patient visits).

Each of these groups was then further subdivided to correlate with the casino-shift scheduling of 10:00 pm to 4:00 am, and 4:00 am to 10:00 am, and analyzed for these time periods. In the casino-shift intervention period, there were 2495 visits (10:00 pm-4:00 am 954 visits, 4:00 am-10:00 am 1541 visits), and in the comparison group 14292 visits (10:00 pm-4:00 am 5678 visits, 4:00 am-10:00 am 8614 visits).

WT

**Figure 1:**
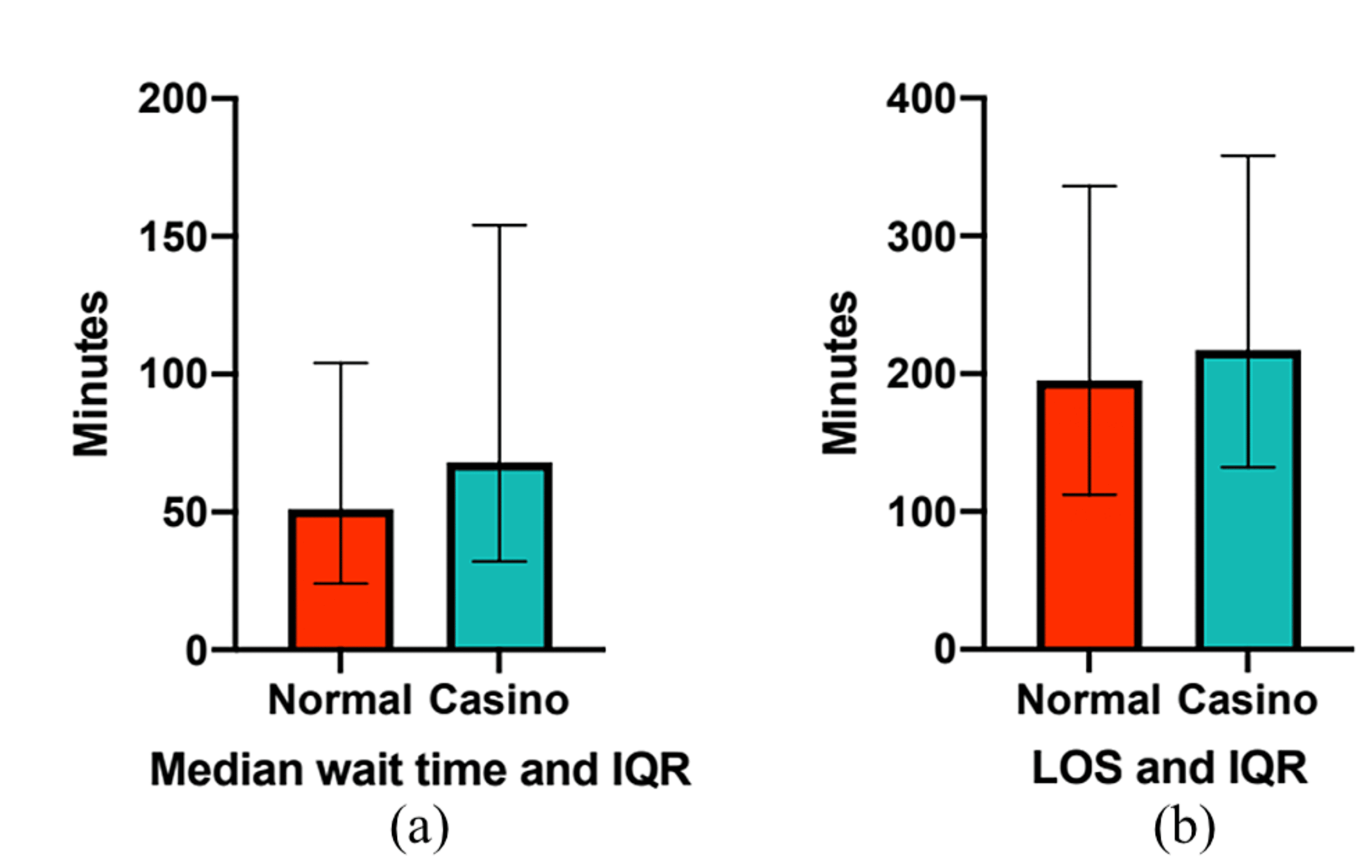
Control group and casino-shift median wait times and length of stay (LOS) with their respective interquartile ranges (IQRs).

**Figure 2:**
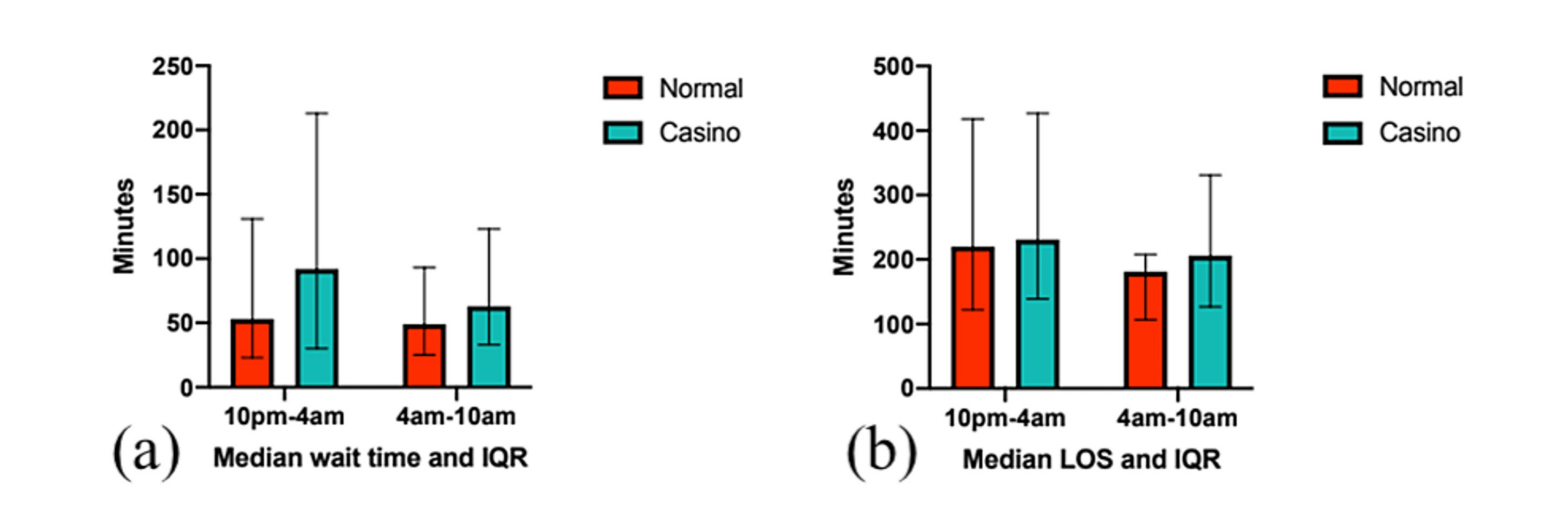
Subgroup analyses of 10:00 pm-4:00 am and 4:00 am-10:00 am for control group and casino-shift median wait times and length of stay (LOS) with their respective interquartile ranges (IQRs).

The overall median WT overnight during the casino-shift intervention period was 68 minutes (IQR 31-154 minutes), as shown in Figure 1a. The subgroup analyses of 10:00 pm-4:00 am and 4:00 am-10:00 am had median WTs of 92 minutes (IQR 30-213 minutes) and 63 minutes (IQR 33-123 minutes), respectively (Figure 2a).

The overall median WT in the comparison “normal” scheduling periods was shorter at 51 minutes (IQR 21-104 minutes) (Figure 1a). In subgroup analyses of 10:00 pm-4:00 am and 4:00 am-10:00 am, the median WTs were 53 minutes (IQR 23-131 minutes) and 49 minutes (IQR 25-93 minutes), respectively (Figure 2a).

LOS

The overall median LOS overnight during the casino-shift intervention period was 217 minutes (IQR 132-358 minutes), shown in Figure 1b. Subgroup analyses of 10:00 pm-4:00 am and 4:00 am-10:00 am had a median LOS of 231 minutes (IQR 139-427 minutes) and 206 minutes (IQR 127-331 minutes) (Figure 2b).

The median LOS overnight in the normal comparison group was shorter at 195 minutes (IQR 112-336 minutes) (Figure 1b). The subgroup analyses of 10:00 pm-4:00 am and 4:00 am-10:00 am had median LOS of 220 minutes (IQR 122-418 minutes) and 181 minutes (IQR 107-208 minutes) (Figure 2b).

Admissions

There were 332 overnight admissions (166 admissions/month) during the two-month casino-shift intervention period: 155 patients were admitted between 10:00 pm and 4:00 am, while 177 were admitted between 4:00 am and 10:00 pm. The rate was lower during the control period, with 1926 overnight admissions (193 admissions/month) during the 10-month comparison period: 899 patients were admitted between 10:00 pm and 4:00 am, while 1027 were admitted between 4:00 am and 10:00 pm.

## Discussion

We assessed the impact that a casino-shift scheduling trial period might have on patient flow indicators. WT and LOS were chosen as patient flow indicators as these are easy to define and can be reliably measured. Other indicators such as the rate of return visits and patient satisfaction were not studied due to many confounding factors and the unreliability of descriptive data.

We found that median WT and LOS were longer during the casino-shift intervention period by an absolute difference of 17 minutes and 22 minutes, respectively. Admission rates were grossly comparable at 166 admissions/month during the two-month casino-shift period compared with 193 admissions/month during the 10-month normal scheduling comparison period.

Despite our hypothesis that by preserving the circadian rhythm in the physicians covering the casino-shift, they would arrive overnight feeling more refreshed and cognitively alert, and thus perform more efficiently, we failed to see this translate into improved patient flow through the department with decreasing WT and LOS. While our results did not support our original hypothesis and the casino-shift model was associated with a statistically non-significant decrease in patient flow measures, there are several factors that may have contributed to these findings.

Patient flow through the ED is impacted by many confounding variables independent of physician workflow [2]. At the departmental level, potential contributors include staffing shortages, rate of patient arrival per hour, complex patient presentations requiring more physician input, and the presentation of simultaneous critically ill patients during single physician coverage periods [10]. Extra-departmental factors impacting patient flow include time to consultation due to specialty delays, patients requiring multiple specialist consultations, delays in obtaining investigations (e.g. CT), delays in the transfer of admissions to accepting units, and boarding of admitted patients in the ED [11].

Limitations of this work include the study being conducted at a single ED which limits generalizability due to the many confounding variables mentioned above. To mitigate this, we attempted to use representative time periods as a control. Subgroup analysis by triage level was not completed, as such we are unable to extrapolate what impact the casino-shift scheduling had on patients within each Canadian Triage Acuity Score (CTAS; categories 1-5). As a result, we cannot ascertain whether the increase in WT and LOS was uniform across all CTAS categories or if the trial period included a higher proportion of high-acuity patients, potentially acting as a confounder. As a retrospective analysis, we are also unable to concretely determine what factors were ongoing in the department that could have contributed to patient flow delays (complexity of patients, delays in consultations, delays in investigations, boarding of admitted patients, staffing levels of nurses, number of medical learners in the department).

Given the detrimental effects that shift work has on physicians and the prevalence of burnout in this population, it is important that administrators continue to consider scheduling alternatives, such as the casino-shift model, that minimize the impact of sleep deprivation on practitioners but optimize department functioning and patient flow [5].

ED patient flow is a complex interaction of multiple variables; it would be interesting to observe objective cognitive function testing of physicians working the casino-shift versus traditional overnight shifts to assess how the maintenance of the anchor period translates to cognitive function and decision-making abilities. Existing literature on casino-shift scheduling in emergency medicine has focused on the impact of the scheduling change on physicians’ preferences and well-being. To our knowledge, this is the first study to examine the effects of a casino-shift schedule on patient flow in the ED.

## Conclusions

In conclusion, this study involving a retrospective analysis of patient flow indicators during a two-month casino-shift intervention period revealed that WT and LOS were longer compared to the traditional overnight shift period. Patient flow in the ED is subject to a number of contributing variables irrespective of the cognitive functioning and performance of the physician. At a time when ED crowding is a national issue, we need to continue looking at scheduling strategies that optimize patient flow and productivity with consideration for physician well-being. As a relatively new concept in emergency medicine scheduling, the casino-shift model warrants further investigation of its impact on patient flow and safety given the known wellness benefits in an occupation prone to burnout. Future research could look at the impact on flow variables in various subgroups, such as by CTAS, normalizing time to assessment by other specialties, and adjusting for staffing levels.
